# Four different fixation strategies for ipsilateral femoral neck and shaft fractures: a finite element analysis

**DOI:** 10.1186/s13018-026-07040-7

**Published:** 2026-07-07

**Authors:** Yuan Liu, Xiang Zhang, Aïcha Noura Yélèen Traoré, Sujan Shakya, Guichen Liang, Yi Li, Xin Duan

**Affiliations:** 1 Department of Orthopaedic Surgery and Orthopedic Research Institute, West China Hospital, Sichuan University, Chengdu, China; 2 Department of Thoracic Surgery, The Affiliated Hospital, Southwest Medical University, Luzhou, China; 3 Department of Clinical Medicine, West China Hospital, Sichuan University, Chengdu, Sichuan China; 4 The Fifth People’s Hospital of Sichuan Province, Chengdu, China

**Keywords:** Ipsilateral femoral neck and shaft fractures, FEA, Internal fixation, Biomechanics, Antegrade intramedullary nail

## Abstract

**Background:**

Ipsilateral femoral neck and shaft fractures are complex injuries that result from high-energy trauma. Despite the availability of multiple fixation strategies, optimal surgical management remains a subject of ongoing debate and comparative biomechanical data are lacking. This study evaluated the stability of four commonly used constructs for internal fixation using patient-specific finite element analysis (FEA) to provide a robust biomechanical rationale for surgical decision-making.

**Methods:**

Patient-specific FEA models were reconstructed using computed tomography imaging data of eight patients to simulate a complex injury pattern involving an ipsilateral Pauwels type III femoral neck fracture combined with a comminuted femoral shaft fracture. Four distinct internal fixation strategies were evaluated: proximal femoral nail antirotation, compression plate with cannulated screws, antegrade intramedullary nail with cannulated screws (AN-CS), and long proximal femoral locking plate. Stability parameters were quantified under simulated axial and torsional loading conditions to assess the mechanical performance of each construct.

**Results:**

Under both loading conditions, the AN-CS group consistently maintained low levels of femoral and implant displacement while demonstrating a more uniform stress distribution and minimum interfragmentary motion at the femoral neck fracture site.

**Conclusion:**

Of the four fixation methods evaluated in this simulation study, the AN-CS construct exhibited the best biomechanical performance overall. The AN-CS fixation effectively balanced interfragmentary stability with a uniform stress distribution, thereby providing a biomechanical basis to guide subsequent clinical decision-making.

**Supplementary Information:**

The online version contains supplementary material available at https://doi.org/10.1186/s13018-026-07040-7.

## Introduction

The incidence of ipsilateral femoral neck and shaft fractures (IFNSF) is low; however, approximately 9% of patients presenting with a femoral shaft fracture also sustain a concurrent femoral neck fracture [1, 2]. These injuries are primarily caused by high-energy trauma mechanisms, such as motor vehicle accidents and falls from a height [3]. However, compared with isolated fractures, these combined injuries are associated with a significantly higher risk of complications, including displacement, nonunion, malunion, and osteonecrosis, which severely compromise the long-term quality of life of patients [4, 5]. With advancements in surgical techniques and fixation strategies, implants, such as intramedullary nails and cannulated screws combined with plates, have been used to treat fractures [6, 7]. Nevertheless, the risk of postoperative complications remains unacceptably high, with reported nonunion rates of 5% for the femoral neck and 20% for the femoral shaft [8]. Furthermore, an initial proximal femoral fracture significantly increases the risk of a subsequent contralateral fracture, potentially worsening exacerbating the overall long-term burden on patients [9].

The overall stability of the fixation construct is a critical risk factor influencing postoperative complications [10]. In patients experiencing IFNSF, the specific fracture patterns of the neck and shaft directly dictate the overall stability achieved after fixation interventions [11]. Furthermore, the choice of fixation strategy is crucial for the management of these injuries. In a long-term follow-up study of patients treated with either single or dual implants, Bastian et al. [12] found that the risk of complications was significantly higher in the dual implant group than in the single implant group. However, research exploring the biomechanical characteristics of different fixation strategies for the IFNSF is currently lacking. A stable biomechanical environment at the fracture site is a key factor for reducing postoperative fixation-related complications and promoting fracture healing [13]. Therefore, an in-depth understanding of the biomechanical characteristics of these fixation methods is of paramount importance in guiding the selection of internal fixation strategies for the surgical management of these complex fractures.

Finite element analysis (FEA) is a numerical method used to simulate physical systems [14]. Owing to its high efficiency and accuracy, this technique has been widely applied in orthopedics and serves as a crucial adjunct to clinical and biomechanical research. Numerous studies have validated the efficacy of FEA in predicting the biomechanical microenvironment of femoral fractures [15]. Therefore, this study used FEA to evaluate the stability of fixation constructs using four implants commonly used for IFNSF. This study aimed to provide a biomechanical basis for the selection of internal surgical fixation strategies.

## Methods

### study participants

This study was approved by the Medical Ethics Committee of our institution (approval no. [2020776]). Full-length femoral computed tomography (CT) images (SOMATOM Definition AS, Siemens, Germany; slice thickness: 0.6 mm) and demographic data (Table 1) for the eight patients with IFNSF were obtained from the institutional database. The three-dimensional (3D) models were constructed based on the imaging data of the intact femurs. None of the patients had a history of hip or systemic diseases at the time of the CT scan.

**Table 1:** Baseline information of participants

	Age	gender	BMI (Kg/cm2)	Young’s modulus: cortical bone(MPa)	Young’s modulus: cancellous bone(MPa)	Injury mechanism
Model 1	56	Man	23.3	22003.66	985.62	Traffic accident injury
Model 2	55	Man	26.0	16138.93	1264.76	Traffic accident injury
Model 3	64	Woman	23.4	16365.24	759.14	injury due to fall
Model 4	58	Man	21.6	15660.87	1270.09	Traffic accident injury
Model 5	37	Man	22.0	9336.74	1284.54	injury by fall from height
Model 6	14	Woman	22.0	12421.75	784.27	injury by fall from height
Model 7	55	Man	24.22	11487.48	977.23	Traffic accident injury
Model 8	70	Woman	24.97	12102.68	832.13	Traffic accident injury

### FEA modeling

All CT images were saved in the Digital Imaging and Communications in Medicine format and imported into Mimics software (v.21.0; Materialize, Leuven, Belgium) for 3D modeling. Subsequently, the femoral 3D models in the STL format were imported into Geomagic Wrap 2021 (Geomagic, USA) for further surface smoothing and fitting. The processed models were exported as STP files. Finally, a model of an ipsilateral Pauwels type III femoral neck fracture combined with a comminuted femoral shaft fracture was constructed using SolidWorks 2021 (Dassault Systèmes, France) [16].

Based on clinical experience at our institution, four clinically common fixation techniques were employed to address IFNSF: proximal femoral nail antirotation (PFNA), compression plate combined with cannulated screws (CP-CS), antegrade intramedullary nail combined with cannulated screws (AN-CS), and long proximal femoral locking plate (LPFLP).

**Fig. 1:**
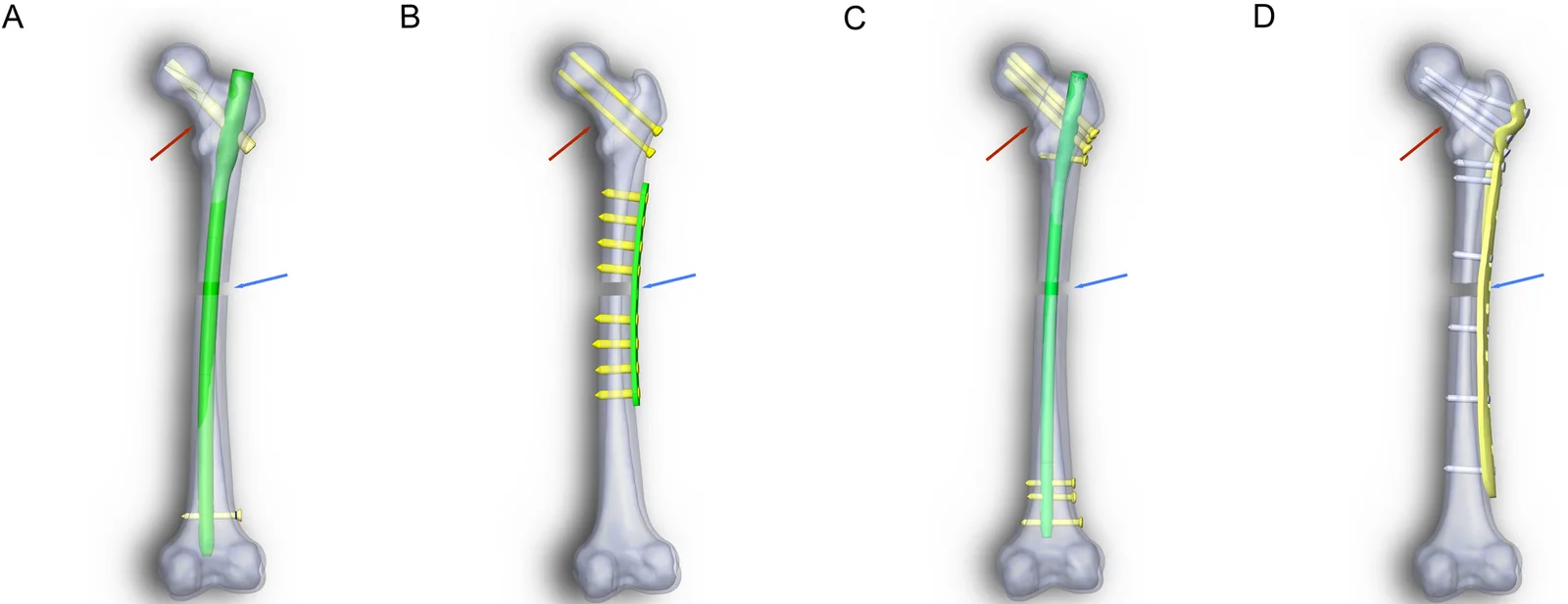
Schematic diagram of ipsilateral femoral neck and shaft fracture models fixed by four clinically common techniques.** A** PFNA** B** CP-CS** C** AN-CS** D** LPFLP

Four clinically applied internal fixation constructs were modeled using specific geometric dimensions and standardized positioning criteria, in accordance with implant specifications and surgical protocols (Fig. 1). In the PFNA group, an 11-mm diameter intramedullary nail was utilized. An 11-mm helical blade was placed in the inferior central aspect of the femoral neck, with its apex positioned 5 mm below the subchondral bone of the femoral head. Distal bicortical fixation was achieved using 5.0-mm locking screws [17]. In the CP-CS group, the compression plate used was a narrow dynamic compression locking plate (ZXSD12) measuring 11 mm in width and 3.0 mm in thickness. Three parallel 3.0-mm cannulated screws were placed in the femoral neck in an “inverted triangular” configuration to provide bicortical fixation. The plate was fixed to the lateral aspect of the femoral shaft to ensure that all screw holes were maintained at a minimum of 5 mm from the fracture line [18]. In the AN-CS group, an 11-mm antegrade nail was introduced via an entry point slightly posterior to the greater trochanteric tip and was centered within the medullary cavity. Next, 3.0-mm cannulated screws were positioned in the anterior third of the femoral neck to stabilize the Pauwels type III fracture line via bicortical fixation [19]. In the LPFLP group, an extended proximal femoral locking plate, consisting of a proximal femoral locking plate integrated with a femoral dynamic compression locking plate, was used. This construct was 17.5 mm wide, 6.0 mm thick, and 120–395 mm long. Bicortical fixation was performed using 6.0-mm diameter locking screws. Following the surgical protocols, the proximal screws were placed within the middle-to-lower third of the femoral neck while carefully bypassing the fracture line [18].

### Finite element parameter settings

The constructed fracture fixation models were imported into the ANSYS Workbench 2021R2 (ANSYS, Inc., Canonsburg, PA, USA) for further FEA. All fracture and internal fixation models were assumed to be isotropic and linearly elastic materials. The apparent density (ρ), Young’s modulus (E), and Poisson’s ratio (* v *) of the cortical and cancellous bone were calculated from the Hounsfield units of the CT images using the following equations:

$$ \rho {\text{(g/cm3) = 0}}{\text{.000968 * HU + 0}}{\mathrm{.5}} $$

$$ {\text{If }}\rho < {\mathrm{1}}.{\text{2 g/cm3}},{\text{E = 2014 }}\rho ^{{{\mathrm{2}}{\mathrm{.5}}}} ({\mathrm{Mpa}}),{\text{ }}v{\text{ = 0}}{\mathrm{.2}}. $$

$$ {\text{If }}\rho {\text{ > 1}}.{\text{2 g/cm3}},{\text{E = 1763}}\rho ^{{{\mathrm{3}}{\mathrm{.2}}}} ({\mathrm{Mpa}}),v{\text{ = 0}}{\mathrm{.32}}{\mathrm{.}} $$

All internal fixation models were assigned the material properties of a titanium alloy (Ti-6Al-7Nb) with a Young’s modulus of 105 GPa, Poisson’s ratio of 0.34, and a yield strength of 800 MPa [20].

Frictional contacts were defined between the fracture surfaces, bone and implants, and implant components with friction coefficients of 0.46, 0.30, and 0.23, respectively. All the other contacts were defined as bonded [21]. All models were meshed using quadratic tetrahedral elements (C3D10). For the mesh convergence analysis, the bone and implant models were meshed with element sizes of 1.0, 2.0, 3.0, 4.0, and 5.0 mm, and 1.0, 1.5, 2, 2.5, and 3.0 mm, respectively. Under simulated axial and torsional loading, mesh sizes of 3 mm for the bone and 2 mm for the implants yielded mesh-independent results (Table 2). The numbers of elements and nodes for all the models are listed in (Table 3).

**Table 2:** Mesh convergence analysis of the maximum von Mises stress in relation to mesh size for implant and bone models

Mesh size	1	2	3	4	5
MIVMS^a^ (MPa)	258.5	256.9	258.7	252.2	257.6
MFVMS ^b^(MPa)	112.5	70.2	59.5	58.6	58.1
Computing time(s)	5640	3060	1406	480	675

Taking PFNA group as an example

^a^MIVMS, maximum implant von Mises stress. ^b^MFVMS, maximum femur von Mises stress

**Table 3:** Number of grids and nodes

	PFNA	CP-CS	AN-CS	LPFLP
Number of nodes	343,122	344,288	392,487	673,682
Number of units	213,614	216,444	248,364	455,865

**Fig. 2:**
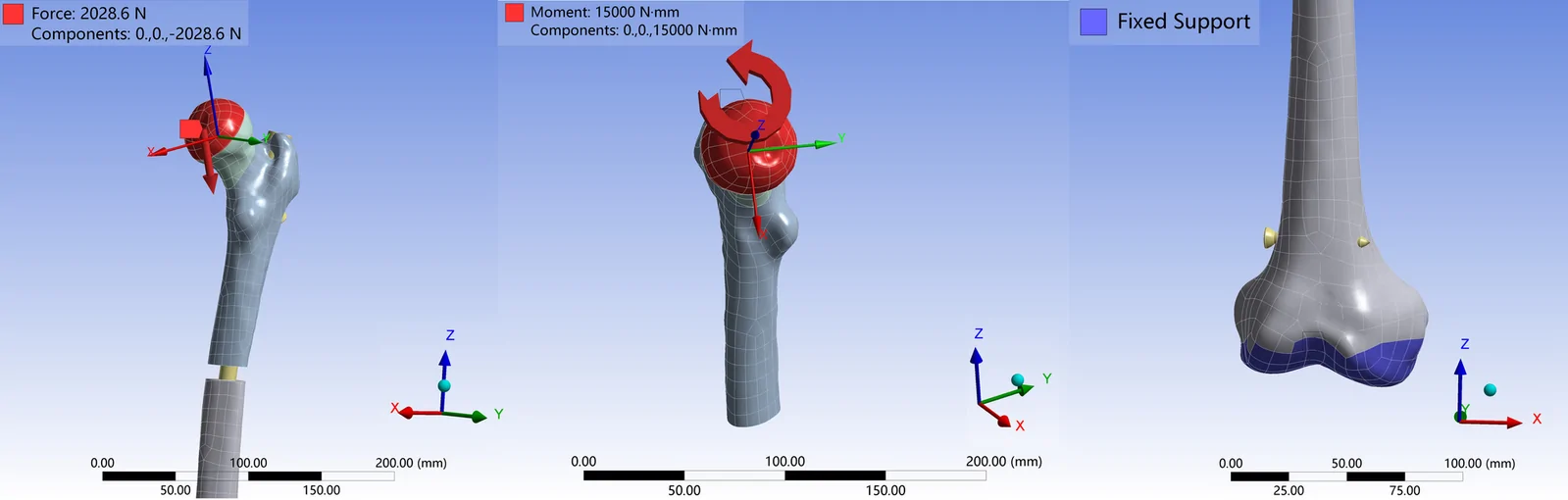
Schematic diagram of loading and boundary conditions applied to the finite element models, using the PFNA group as an example.** A** Axial load simulating single-leg stance** B** Maximum torsional load during daily activities** C** Boundary conditions of the model

This study simulated the loading conditions experienced by patients during postoperative single-leg stance and performance of daily activities. The degrees of freedom of the distal femur were constrained to zero in the X, Y, and Z directions [22]. A load equivalent to 300% of the patient’s body weight was applied to the femoral head-acetabulum contact surface along the mechanical axis of the femur. This loading configuration represents the force sustained by the femoral head during the single-leg stance phase of normal gait [23]. A torsional load of 15 N.m was applied to the surface of the femoral head along the axis of the femoral neck, which represents the maximum torsional load exerted on the femoral head during normal human gait [24] (Fig. 2).

### Assessment of biomechanical parameters

**Fig. 3:**
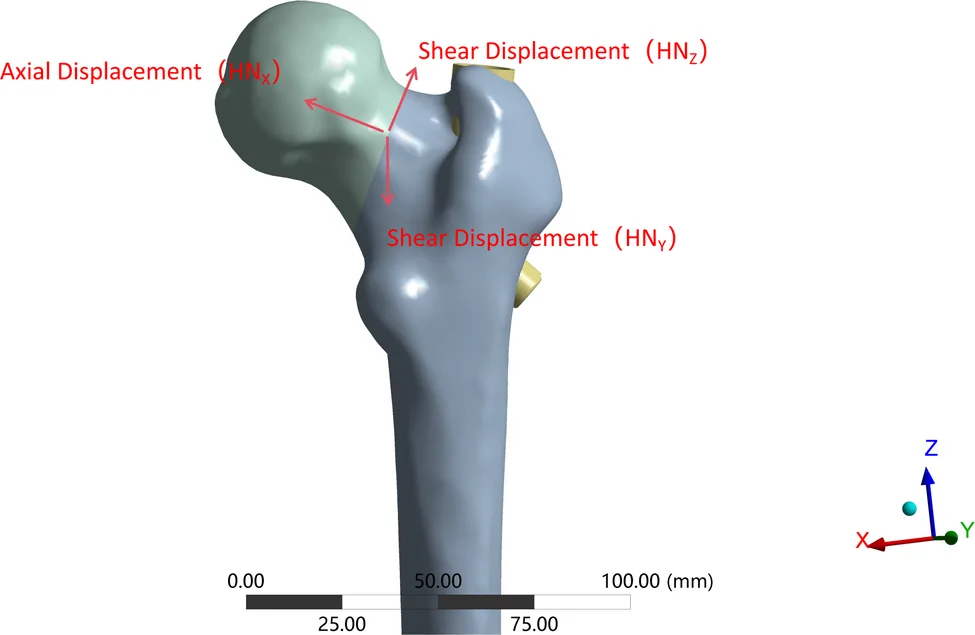
Schematic diagram illustrating the maximum femoral head-neck interfragmentary motion (MFHNIM), which is determined by evaluating the relative displacement between the femoral head and the femoral neck fracture fragment along the X, Y, and Z axes

Based on previous studies [25, 26], we evaluated six biomechanical parameters related to the fixation stability of IFNSF: the maximum implant von Mises stress (MIVMS), maximum implant displacement (MID), maximum proximal femoral von Mises stress (MPFVMS), maximum femoral von Mises stress (MFVMS), maximum femoral displacement (MFD), and maximum femoral head and neck interfragmentary motion (MFHNIM). MFHNIM was calculated using the following equation:

$$ {\text{MFHNIM = }}\sqrt {{\mathrm{HN}}_{{\mathrm{X}}}^{{\mathrm{2}}} {\text{ + HN}}_{{\mathrm{Y}}}^{{\mathrm{2}}} {\text{ + HN}}_{{\mathrm{Z}}}^{{\mathrm{2}}} } $$

where, HNX, HNY, and HNZ represent the relative displacements of the femoral head with respect to the femoral neck along the X, Y, and Z axes, respectively [27] (Fig. 3).

### Statistical analysis

Statistical analyses of all simulation results were performed using SPSS (version 22.0 IBM Corp., Armonk, NY, USA) and GraphPad Prism 10.4 (GraphPad Software, San Diego, CA, USA). The simulation results are expressed as the mean ± standard deviation (SD). Notably, this SD reflects the intragroup variance originating from anatomical differences among the patient models rather than repeated experimental measurement errors. Analysis of variance was used to compare the biomechanical data among the groups across the same batch of models. Normality and homogeneity of variance were verified using the Shapiro–Wilk and Levene’s tests, respectively. A *P*-value < 0.05 was considered statistically significant.

## Result

### Femoral von Mises stress

**Fig. 4:**
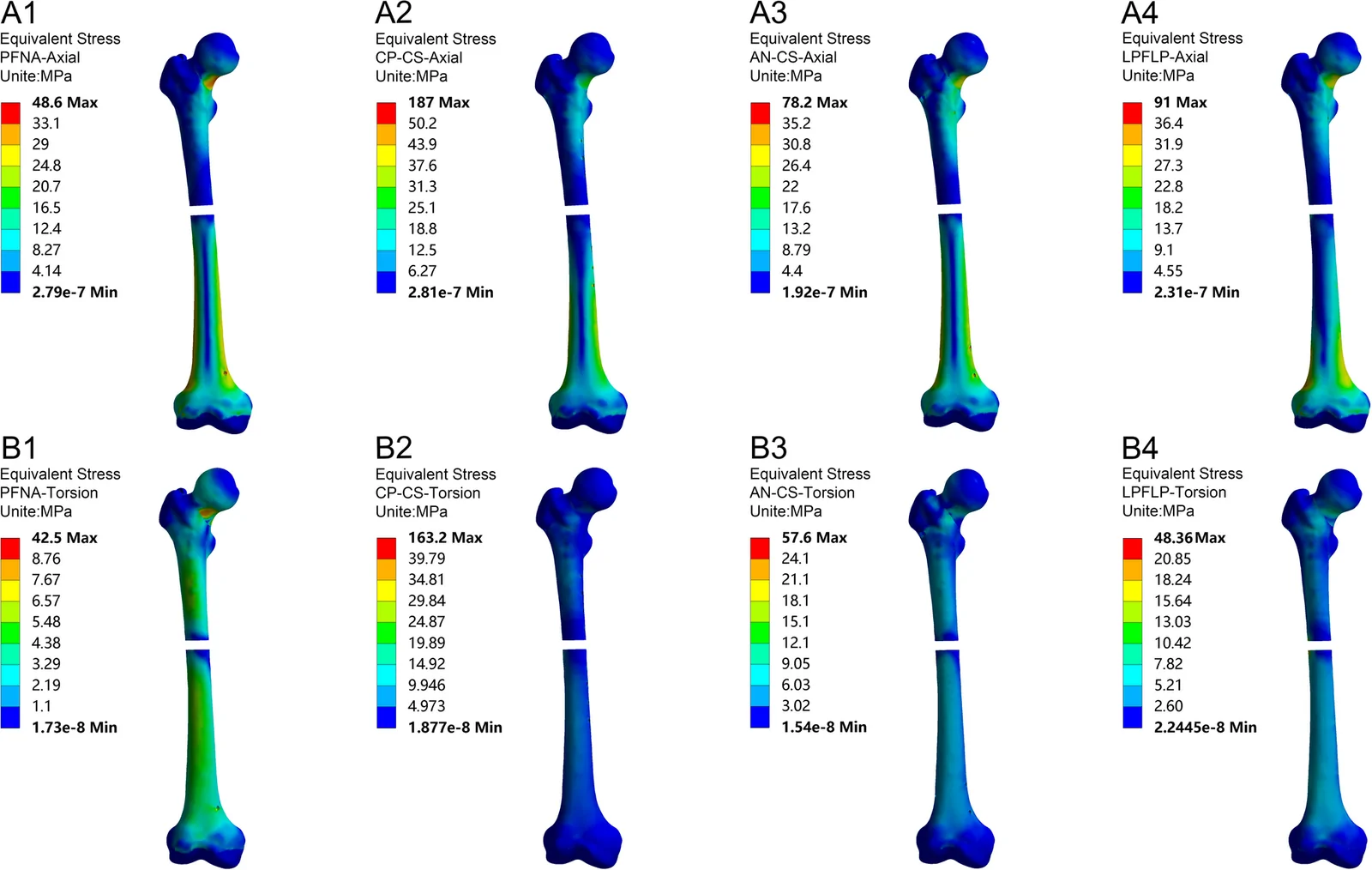
Contour plots of the maximum femur von Mises stress (MFVMS).** A** Under axial loading** B** Under torsional loading (1) PFNA (2) CP-CS (3) AN-CS (4) LPFLP

Under axial loading, the CP-CS group exhibited the highest MFVMS (182.7 ± 29.4 MPa), with stress concentrated at the site of the distal femoral locking screws. In contrast, the PFNA group demonstrated the lowest MFVMS (51.9 ± 5.0 MPa), representing a 71.6% reduction compared with the CP-CS group. In the AN-CS group (80.6 ± 7.2 MPa), MFVMS was similarly concentrated at the distal femoral locking screws, whereas in the LPFLP group (86.8 ± 9.8 MPa), stress was concentrated at the inferior aspect of the medial femoral neck cortex. No statistically significant differences were observed between these two groups (*P* > 0.05) (Fig. 4A, Supplementary Fig. 1A). Under torsional loading, the stress distribution trends were consistent with those observed under axial loading. The CP-CS group remained the highest (172.0 ± 33.6 MPa), with stress concentrations located at the comminuted fracture site of the femoral-mid shaft. The PFNA group showed the lowest MFVMS (43.8 ± 2.2 MPa), a 74.5% reduction compared with the CP-CS group, with stress concentrated at the inferior aspect of the medial femoral neck cortex. In the AN-CS group (56.3 ± 6.7 MPa), stress was concentrated at the anteroinferior aspect of the medial femoral neck cortex, whereas in the LPFLP group (47.5 ± 6.5 MPa), stress was concentrated at the posteroinferior aspect. No significant differences were found between the two groups and the PFNA group (*P* > 0.05) (Fig. 4B, Supplementary Fig. 1B).

### Implant von Mises stress

**Fig. 5:**
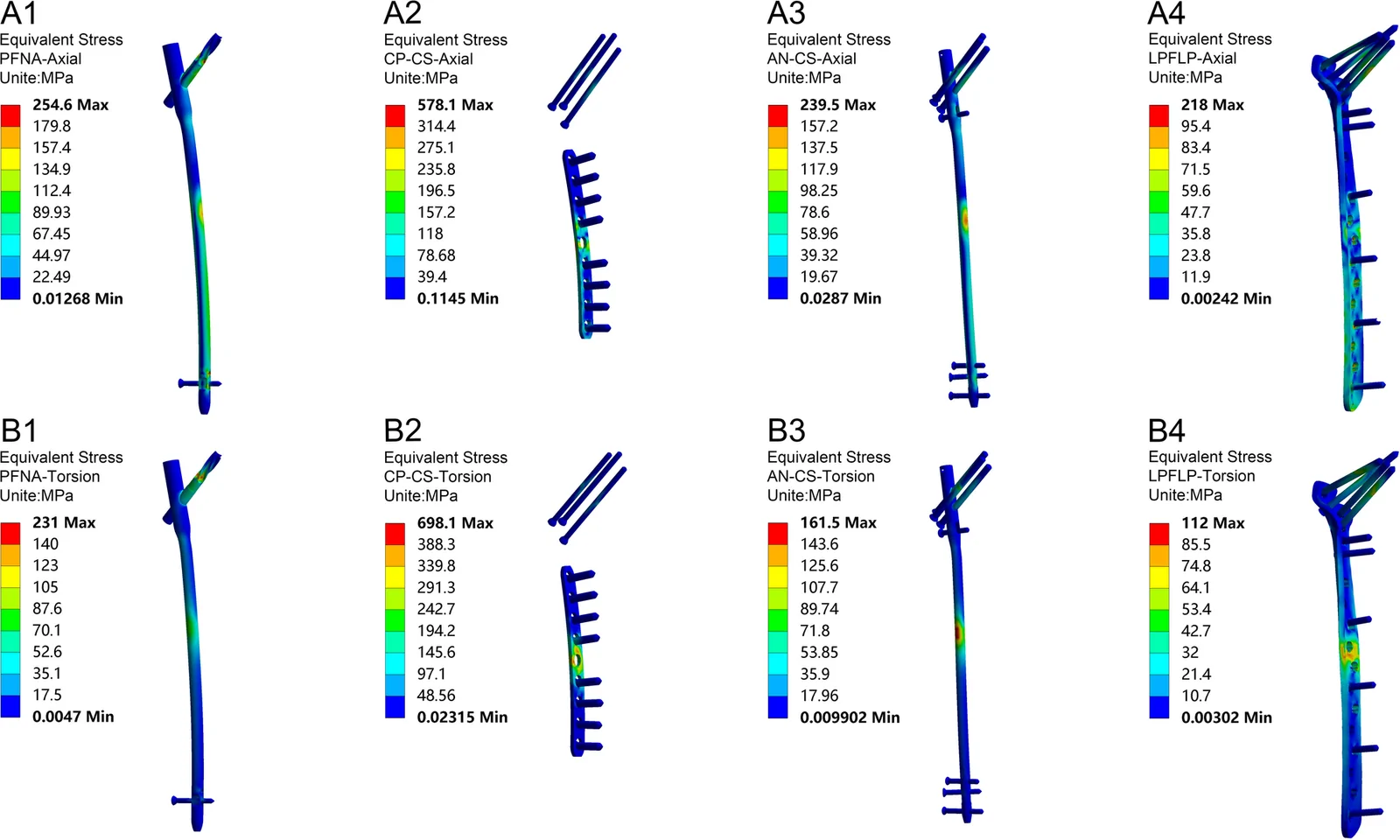
Contour plots of the maximum implant von Mises stress(MIVMS).** A** Under axial loading** B** Under torsional loading (1) PFNA (2) CP-CS (3) AN-CS (4) LPFLP

Under axial loading, the CP-CS group exhibited the highest MIVMS (603.1 ± 46.3 MPa), with stress concentrated on the surface of the middle segment of the plate at the site of the comminuted femoral shaft fracture. The LPFLP group showed the lowest MIVMS (242.9 ± 21.6 MPa), representing a 59.7% reduction compared with the CP-CS group; stress was similarly concentrated at the middle segment of the plate corresponding to the fracture site. In the AN-CS group (249.8 ± 20.5 MPa), stress was concentrated at the middle segment of the intramedullary nail corresponding to the fracture site, whereas in the PFNA group (253.0 ± 30.9 MPa), it was concentrated at the middle segment of the helical blade (at the femoral neck fracture line). No statistically significant differences were observed between the three groups (*P* > 0.05) (Fig. 5A,Supplementary Fig. 2A). Under torsional loading, the CP-CS group continued to exhibit the highest MIVMS (735.0 ± 86.5 MPa) with a stress concentration at a site consistent with that observed under axial loading. The LPFLP group demonstrated the lowest MIVMS (115.6 ± 7.8 MPa), an 84.2% reduction compared with the CP-CS group, with stress concentrated at the middle segment of the locking screw at the femoral neck. In the PFNA group (227.0 ± 11.5 MPa), stress was concentrated on the surface of the helical blade (at the femoral neck fracture line), whereas in the AN-CS group (161.1 ± 1.1 MPa), stress was concentrated at the middle segment of the intramedullary nail corresponding to the fracture site. The MIVMS in both the PFNA and AN-CS groups was significantly lower than that observed in the CP-CS group (*P* < 0.05) (Fig. 5B, Supplementary Fig. 2B).

### Proximal femoral von Mises stress

**Fig. 6:**
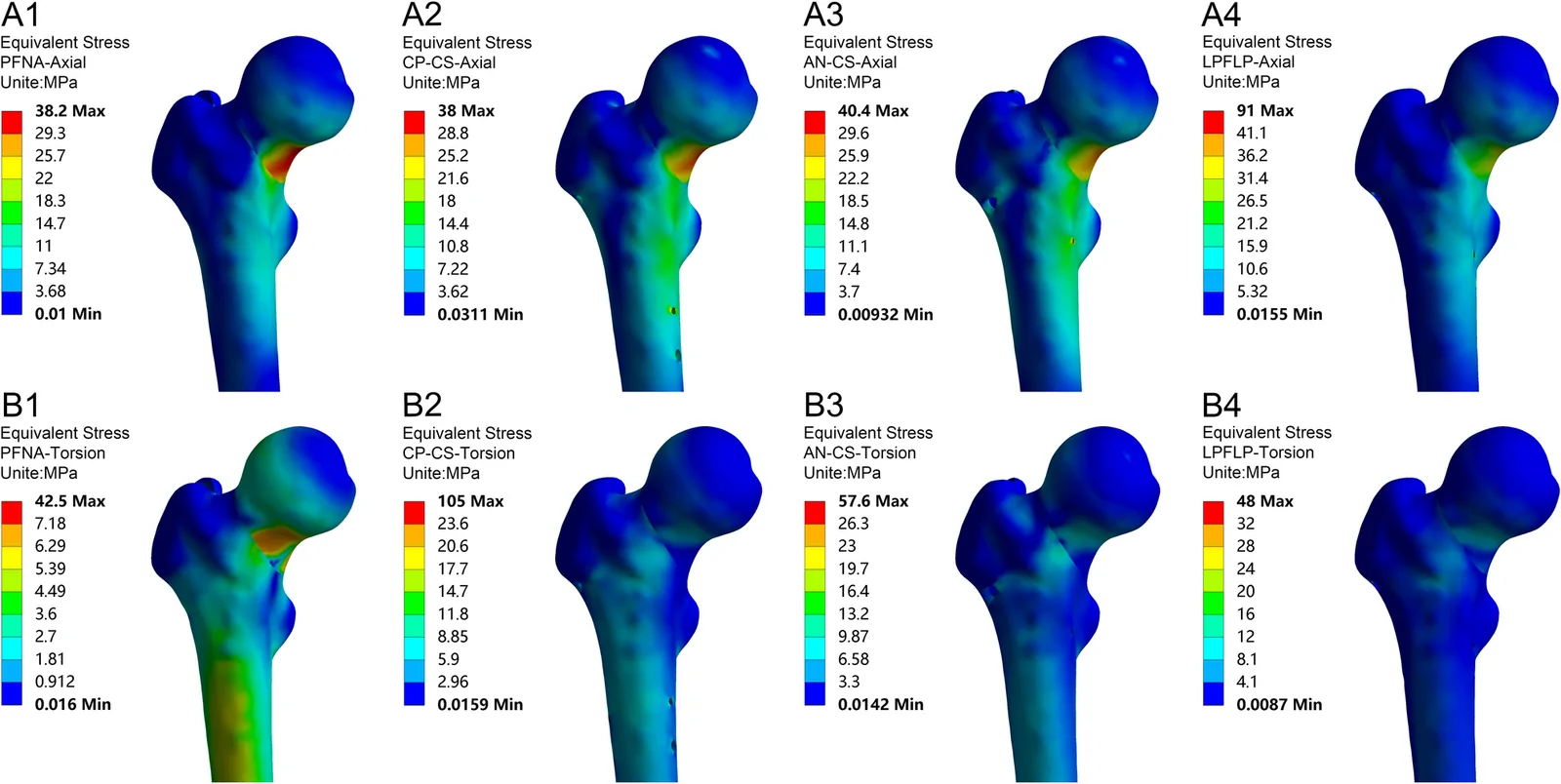
Contour plots of the maximum proximal femur von Mises stress(MPFVMS).** A** Under axial loading** B** Under torsional loading (1) PFNA (2) CP-CS (3) AN-CS (4) LPFLP

Under axial loading, the LPFLP group exhibited the highest MPFVMS (83.2 ± 13.2 MPa), whereas the PFNA group showed the lowest (41.1 ± 6.0 MPa), representing a 50.6% reduction compared with the LPFLP group. The stress levels in the AN-CS group (44.1 ± 8.3 MPa) and the CP-CS group (41.1 ± 6.4 MPa) were similar, and no significant differences were observed across these three groups (PFNA, AN-CS, and CP-CS) (*P* > 0.05) (Fig. 6A, Supplementary Fig. 3A). Under torsional loading, the CP-CS group displayed the highest MPFVMS (104.1 ± 9.6 MPa), whereas the PFNA group exhibited the lowest (43.8 ± 2.3 MPa), representing a 57.9% reduction compared with the CP-CS group. No significant difference in MPFVMS was found between the AN-CS group (56.3 ± 6.7 MPa) and the LPFLP group (47.5 ± 6.5 MPa) (*P* > 0.05), yet both were significantly lower than the CP-CS group (*P* < 0.05). The maximum stress was primarily concentrated at the inferior aspect of the medial femoral neck cortex (Fig. 6B, Supplementary Fig. 3B).

### Femoral displacement

**Fig. 7:**
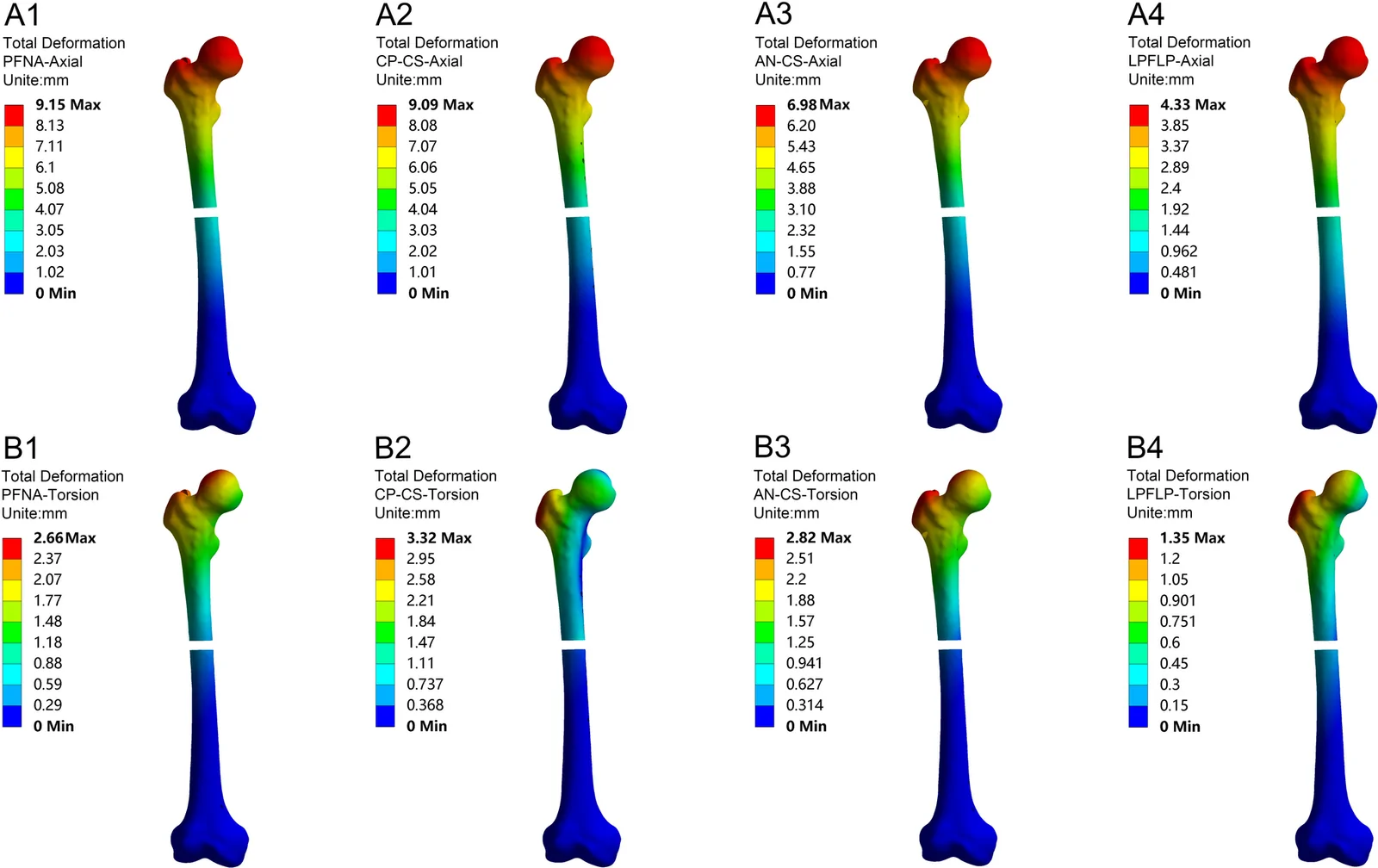
Contour plots of the maximum femur displacement(MFD).** A** Under axial loading** B** Under torsional loading (1) PFNA (2) CP-CS (3) AN-CS (4) LPFLP

Under axial loading, the CP-CS group exhibited the highest MFD (10.4 ± 1.7 mm), whereas the LPFLP group showed the lowest (5.1 ± 0.8 mm), representing a 51.0% reduction compared with the CP-CS group. The MFD values for the AN-CS and PFNA groups fell between these extremes, with no significant differences observed between them (*P* > 0.05) (Fig. 7A, Supplementary Fig. 4A). Under torsional loading, the displacement distribution trend was consistent with that observed under axial loading. The CP-CS group demonstrated the highest MFD (3.5 ± 0.4 mm), whereas the LPFLP group exhibited the lowest (1.5 ± 0.2 mm), a 57.1% reduction compared with the CP-CS group. No significant differences were observed in the displacement levels between the two groups (*P* > 0.05) (Fig. 7B, Supplementary Fig. 4B).

### Implant displacement

**Fig. 8:**
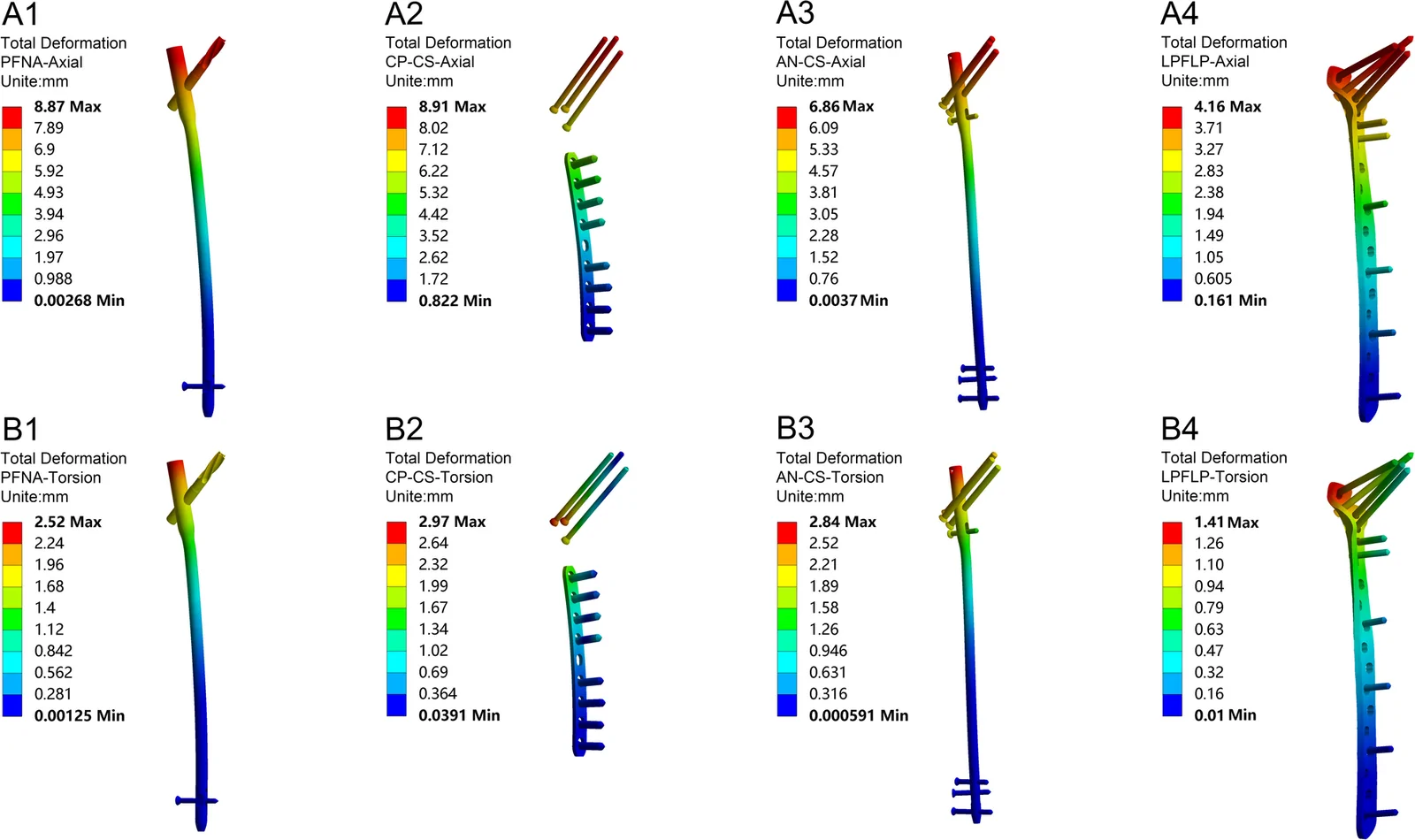
Contour plots of the maximum implant displacement(MID).** A** Under axial loading** B** Under torsional loading (1) PFNA (2) CP-CS (3) AN-CS (4) LPFLP

Under axial loading, the CP-CS group exhibited the highest MID (10.2 ± 1.7 mm), whereas the LPFLP group showed the lowest (4.8 ± 0.8 mm), representing a 52.9% reduction compared with the CP-CS group. No significant difference in the MID was observed between the AN-CS and PFNA groups (*P* > 0.05) (Fig. 8A, Supplementary Fig. 5A). Under torsional loading, the CP-CS group demonstrated the highest MID (3.2 ± 0.4 mm), whereas the LPFLP group displayed the lowest (1.6 ± 0.3 mm), a 50.0% reduction compared with the CP-CS group. No significant differences were observed between the remaining two groups (Fig. 8B, Supplementary Fig. 5B).

### Femoral head and neck interfragmentary motion

Under axial loading, the PFNA group exhibited the highest MFHNIM (0.18 ± 0.01 mm), whereas the LPFLP group showed the lowest (0.098 ± 0.008 mm), representing a 45.6% reduction compared with the PFNA group. No significant difference was found between the AN-CS and CP-CS groups (*P* > 0.05). Under torsional loading, the PFNA group demonstrated the highest MFHNIM (0.29 ± 0.03 mm), whereas the AN-CS group exhibited the lowest (0.12 ± 0.013 mm), representing a 58.6% reduction compared with the PFNA group. Furthermore, no significant differences were observed between the AN-CS group and the other two groups (*P* > 0.05) (Table 4, Supplementary Fig. 6).

**Table 4:** Maximum femoral head-neck interfragmentary motion (MFHNIM) across treatment groups

MFHNIM(mm)	PFNA	CP-CS	AN-CS	LPFLP
Axial	0.181 ± 0.015	0.111 ± 0.010	0.103 ± 0.009	0.098 ± 0.008
Torsion	0.292 ± 0.027	0.132 ± 0.015	0.123 ± 0.013	0.153 ± 0.018

## Discussion

IFNSF is a complex injury typically caused by high-energy trauma. Although the incidence of these fractures is low, the choice of treatment strategy remains controversial owing to the simultaneous involvement of dual anatomical structures, such as the proximal femur and femoral shaft [28]. Unlike fragile proximal femoral fractures, in which clinical mortality is dictated by systemic health factors, IFNSF imposes a higher demand on the biomechanical stability of the fixation construct [29]. Furthermore, the fracture lines span both the weight-bearing zone of the femoral neck and load transfer zone of the femoral shaft. Consequently, effective fixation requires a balance between the antirotational stability of the femoral neck and the axial load-transfer capability of the femoral shaft [30]. In this study, we compared four common internal fixation methods to quantify the biomechanical characteristics using FEA. Our preliminary results indicated that of the four different fixation models, the AN-CS construct exhibited the most favorable overall biomechanical performance.

### Biomechanical validation

To validate the reliability of the finite element model, the simulation results were compared with biomechanical data from previous studies. The stiffness value of the ipsilateral femoral neck and shaft fracture model obtained from the simulation was 324.84 N/mm. When compared with the biomechanical data reported by Wu et al. [31] (344.83 N/mm) under identical boundary and loading conditions, the deviation fell within ± 1 SD. Furthermore, we constructed load-displacement curves and compared them with those of previous studies. The slope error was within 5%, confirming that the model was suitable for subsequent analyses (Supplementary Fig. 7).

### Association between displacement behavior and stability of the four fixation methods

AAccording to the core biomechanical principles of fracture healing, under identical loading conditions, displacement of the fixation system is a critical indicator of fixation construct stability [32, 33]. Greater displacement implies a higher risk of postoperative re-displacement, nonunion, or even malunion. The results of this study revealed significant differences in the displacement distribution among the four fixation methods. The LPFLP group exhibited the lowest femoral and implant displacements under both axial and torsional loadings, whereas the CP-CS group exhibited the highest displacement. The displacement levels in the AN-CS group were intermediate and showed no significant differences compared with those in the LPFLP group. This finding aligned with the results of Tai et al. [34], which suggested that fixation with multiple screws can enhance the overall stability through load sharing. Moreover, appropriate axial micromotion at the fracture line promotes callus formation. Furthermore, an axial micromotion of < 1 mm significantly facilitates fracture healing [35, 36]. Conversely, recent findings have revealed that micromotion in the shear direction is detrimental to callus development and subsequently increases the risk of nonunion. Therefore, effective control of shear micromotion is essential for clinical fracture management [37]. Thus, in this study, the PFNA group exhibited the highest MFHNIM under both axial and torsional loadings conditions, whereas the LPFLP and AN-CS groups exhibited the lowest MFHNIM under axial and torsional loading conditions, respectively. This finding corroborates the inference from previous studies, which reported that the use of additional screws can significantly increase the stability of the overall construct, which is conducive to fracture healing [38]. However, the suboptimal overall performance of the PFNA group in this study may be attributed to technical limitations. For instance, the finite element model could not simulate the compaction effect of the helical blade on the cancellous bone. Therefore, in clinical practice, PFNA may provide better stability and antirotational control of the femoral neck fracture gap [39]. Overall, the stable fixation of the fracture ends was achieved in the LPFLP group using rigid fixation with multiple locking screws and plates. However, in the AN-CS group, the combination of the antegrade intramedullary nail and cannulated screws not only restricted interfragmentary micromotion of the femoral neck via the compression effect of the screws but also resisted axial displacement of the comminuted femoral shaft fracture through the central fixation of the intramedullary nail [19]. Its construct stability was superior to that of the CP-CS and PFNA groups, compensating for the lack of a central load-transfer path in the CP-CS group and the insufficient control of comminuted femoral shaft fracture displacement in the PFNA group. Thus, in the present finite element simulation, the AN-CS construct achieved a more reliable biomechanical performance than the other fixation methods.

### Stress characteristics of various fixation methods

The stress distribution is a core indicator of the effectiveness of fracture constructs fixed with different implants [40]. Under identical loading conditions, more pronounced stress concentration suggests a higher risk of complications such as fixation failure and nonunion [41].

In terms of the MFVMS results, the CP-CS group exhibited the highest values under both axial and torsional loadings, with stress concentrated at the distal femoral locking screws. This was attributed to the eccentric fixation design of the compression plate [42]. Furthermore, because the plate was positioned on the lateral aspect of the femoral shaft, central load transfer was not achieved, resulting in an uneven stress distribution at the fracture site, which may increase the risk of secondary femoral shaft fractures [43]. Under axial loading, the PFNA group demonstrated the lowest femoral stress; however, displacement at the femoral neck fracture gap was substantial. This suggests that although PFNA offers significant advantages in maintaining the stability of the femoral shaft fracture, its stabilizing effect on the femoral neck fracture is limited. Under both axial and torsional loading, the femoral stress in the AN-CS group was comparable to that observed in the LPFLP group, with stress concentrated at the medial cortex of the femoral neck, consistent with the normal physiological load transmission pathway of the femur. The LPFLP group exhibited the highest stress in the proximal femur under axial loading, whereas the CP-CS group exhibited the highest stress under torsional loading. Conversely, the AN-CS group consistently maintained lower stress levels. This difference arises from the unique fixation characteristics of AN-CS: the femoral neck fracture receives compression from the cannulated screws, and the femoral shaft fracture benefits from central fixation. This configuration avoids stress concentration associated with multiple screw insertions and compensates for the biomechanical deficiencies of eccentric fixation [19].

Implant stress directly determines the risk of implant failure. The maximum implant stress observed in the CP-CS group was significantly higher than that in the other three groups under both axial and torsional loading. This was attributed to the rigid structure of the compression plate, which cannot effectively disperse loads at the comminuted fracture site, resulting in stress concentrated primarily in the middle segment of the plate [44]. In clinical practice, the use of the CP-CS construct for IFNSF can increase the risk of internal fixation failure, subsequently increasing the likelihood of revision surgery. In comparison, although the LPFLP group exhibited a lower maximum implant stress, the potential risk of stress concentration in the proximal femur warrants attention. A discrepancy between the clinical outcomes of LPFLP and simulation results has been reported, indicating a higher incidence of femoral neck complications in clinical practice [45].Furthermore, when utilizing a multiple locking screw system, proximal fixation cutout or failure occurs because of stress concentration, amd the resulting severe damage to the femoral anatomy further increases the difficulty of subsequent revision hip arthroplasty [46]. Therefore, LPFLP should be carefully considered in cases of concurrent unstable femoral neck fractures. In contrast, the AN-CS group exhibited uniform stress distribution across the internal fixation construct, which may reduce the risk of complications, such as implant failure, in clinical practice.

Based on a comprehensive comparative analysis of the stress and displacement results, AN-CS demonstrated a stability comparable to that of LPFLP, while maintaining a more uniform stress distribution. Despite providing stable fixation for concurrent IFNSF, the finite element simulation observed in this study indicated that the AN-CS construct achieved superior comprehensive biomechanical performance compared with the other three fixation methods. In contrast, the CP-CS group had a high risk of implant failure because of significant stress concentration and large displacements [47]. Rechter et al. reported a postoperative failure rate as high as 33.3% for multiple cannulated screw fixations for the treatment of IFNSF [48]. Consequently, the CP-CS approach may not be the optimal fixation method for these fractures. Although the LPFLP exhibits good biomechanical stability, stress concentration in the proximal femur may increase the risk of femoral neck complications. Finally, PFNA may be more suitable for patients with stable femoral neck fractures.

### Limitations

This study had certain limitations that should be acknowledged. First, finite element models assume that cancellous and cortical bone are homogeneous, isotropic, and linearly elastic materials. Furthermore, the mechanical contributions of soft tissues such as ligaments and muscles were not incorporated in the analysis. These simplifications may result in slightly higher overall displacement and stress levels than those under actual physiological conditions. Second, this study did not investigate the influence of factors such as varying degrees of fracture comminution or bone mineral density on fixation efficacy. Additionally, although efforts were made to maximize the sample size, the number of included patients remained small. Therefore, the generalizability of the conclusions requires further verification in larger patient cohorts. Third, our study relied on FEA using CT-based reconstruction models. All internal fixation devices were implanted in an idealized virtual environment, whereas the actual surgical procedures were significantly more complex. Fourth, the current simulation did not incorporate cyclic loading conditions or model biological healing processes. Furthermore, the conclusions of this study were derived solely from finite element biomechanical simulations, and large-scale clinical studies are required for further validation. Finally, the current finite element models lacked direct biomechanical validation and were only indirectly compared with previous biomechanical models. This approach failed to fully verify the predictive accuracy of all construct configurations. Moreover, mesh convergence analysis was conducted solely on a representative model; however, different fixation methods may induce local stress variations, potentially leading to differences in mesh sensitivity in specific regions.

## Conclusion

In this study, the biomechanical performance of four clinically common fixation methods for the treatment of IFNSF was systematically evaluated under axial and torsional loading conditions using patient-specific finite element modeling based on CT images. Overall, AN-CS exhibited superior biomechanical performance, suggesting that it may be a promising candidate for further clinical validation.

## Supplementary Information

Below is the link to the electronic supplementary material.

Supplementary Material 1.

## Data Availability

The datasets used and/or analysed during the current study are available from the corresponding author on reasonable request.

## Abbreviations

IFNSF: Ipsilateral femoral neck and shaft fractures
FEA: Finite element analysis
CP-CS: Compression plate combined with cannulated screws
CP-CS: Antegrade intramedullary nail combined with cannulated screws
LPFLP: Long proximal femoral locking plate
MIVMS: Maximum implant von Mises stress
MID: Maximum implant displacement
MPFVMS: Maximum proximal femoral von Mises stress
MFVMS: Maximum femoral von Mises stress
MFD: Maximum femoral displacement
MFHNIM: Maximum femoral head and neck interfragmentary motion
