# Supplementary material for: Four different fixation strategies for ipsilateral femoral neck and shaft fractures: a finite element analysis
Source: J Orthop Surg Res. 2026 Jul 7;21:570. doi: 10.1186/s13018-026-07040-7 (PMC13625483; doi:10.1186/s13018-026-07040-7)
Supplement: Supplementary file 1 [file 13018_2026_7040_MOESM1_ESM.docx]

**Supplementary Figure 1.** The result of maximum femoral von Mises stress(MFVMS).(A) Under axial loading. (B) Under torsional loading.

**Supplementary Figure 2.** The result of maximum Implant von Mises stress(MIVMS).(A) Under axial loading. (B) Under torsional loading.

**Supplementary Figure 3.** The result of maximum proximal femoral von Mises stress(MPFVMS).(A) Under axial loading. (B) Under torsional loading.

**Supplementary Figure 4.** The result of maximum femoral displacement(MFD).(A) Under axial loading. (B) Under torsional loading.

**Supplementary Figure 5.** The result of maximum Implant displacement(MID).(A) Under axial loading. (B) Under torsional loading.

**Supplementary Figure 6.** The result of maximum femoral head and neck interfragmentary motion

(MFHNIM).(A) Under axial loading. (B) Under torsional loading.

**Supplementary Figure 7.** The results demonstrate a comparison of load-displacement curves between the previous research and the validated current model. (A) Reference data from previous study. (B) Experimental data from current study

**Supplementary Table 1** Results of Various Stress Parameters in Each Group

| **Parameters** | | **Groups** | | | | **P-value*** |
| --- | --- | --- | --- | --- | --- | --- |
|  |  | PFNA | CP-CS | AN-CS | LPFLP |  |
| **Axial loading** | MFVMS | 51.9±5.0 | 182.7±29.4 | 80.6±7.2 | 80.8±9.8 | <0.0001 |
|  | MIVMS | 253.0±30.9 | 603.1±46.3 | 249.8±20.5 | 242.9±21.6 | 0.0005 |
|  | MPFVMS | 41.1±6.0 | 41.1±6.4 | 44.1±8.3 | 83.2±13.2 | <0.0001 |
| **Torsional loading** | MFVMS | 43.8±2.3 | 172.0±33.6 | 56.3±6.7 | 47.5±6.5 | <0.0001 |
|  | MIVMS | 227.1±11.5 | 735.0±86.5 | 161.1±1.1 | 115.6±7.8 | <0.0001 |
|  | MPFVMS | 43.8±2.3 | 104.0±9.2 | 56.3±6.7 | 47.5±6.5 | <0.0001 |
